# Temperature patterns in suspected pediatric acute appendicitis

**DOI:** 10.1007/s00383-026-06554-9

**Published:** 2026-08-26

**Authors:** Javier Arredondo Montero, Andrea Herreras Martínez, Luis Rello, Alicia Escudero Villafañe, Marina Iglesias Oricheta, Maria del Mar Larrea Ortiz-Quintana, Lucía Fernández Rodríguez, Pablo Aguado Roncero, María Carmen Campos Calleja, Ricardo Díez, Samuel Sáez Álvarez, Carmen Ruiz de la Cuesta Martín, Carlos Delgado Miguel, Rafael Fernández Atuan

**Affiliations:** 1 Pediatric Surgery Department, Complejo Asistencial Universitario de León, c/Altos de Nava s/n, 24008 León, Castilla y León Spain; 2 Instituto de Investigación Biosanitaria de León (IBIOLEON), León, Spain; 3 Pediatrics Department, Complejo Asistencial Universitario de León, León, Spain; 4 Department of Clinical Biochemistry, Hospital Universitario Miguel Servet, Zaragoza, Spain; 5 Pediatric Surgery Department, Hospital Universitario Fundación Jiménez Díaz, Madrid, Spain; 6 Pediatrics Department, Hospital Universitario Miguel Servet, Zaragoza, Spain; 7 Pathology Department, Complejo Asistencial Universitario de León, León, Spain; 8 Institute for Health Research IdiPAZ, Hospital Universitario La Paz, Madrid, Spain; 9 Pediatric Surgery Department, Hospital Universitario Miguel Servet, Zaragoza, Spain; 10 Surgery Department, Universidad de Zaragoza, Zaragoza, Spain; 11 Aragon Health Research Institute, Hospital Universitario Miguel Servet, Zaragoza, Spain

**Keywords:** Pediatric acute appendicitis, Temperature, Fever, Quadratic modeling, Restricted cubic splines, Continuous predictor, Functional form

## Abstract

**Background:**

Temperature is routinely considered in children with suspected acute appendicitis (AA), but classic appendicitis scores usually reduce it to a binary fever item. This study aimed to evaluate whether continuous temperature measurements contain diagnostic or severity-related information that may be obscured by conventional fever thresholds.

**Methods:**

We performed a secondary exploratory analysis of the BIDIAP 2 (BIomarkers for the DIagnosis of Appendicitis in Pediatrics 2) cohort, a prospective multicenter diagnostic cohort of children evaluated for suspected AA. Two continuous temperature variables were assessed: maximum temperature reported at home before Emergency Department (ED) presentation and temperature measured at ED admission. Single-predictor logistic models evaluated temperature as a linear term, a quadratic term, and a restricted cubic spline. Apparent area under the receiver operating characteristic curve (AUC) estimates with 95% confidence intervals (CIs), likelihood-ratio tests, bootstrap optimism correction, and literature-based fever thresholds were examined.

**Results:**

For AA versus non-surgical abdominal pain (NSAP), maximum home temperature showed no apparent discrimination when modeled linearly (AUC, 0.494; 95% CI, 0.433–0.554), whereas quadratic and spline models yielded higher apparent AUCs of 0.617, with the highest estimated probability of AA in the low-grade fever range. ED temperature showed a similar but weaker pattern, with AUC increasing from 0.591 in the linear model to 0.613 with quadratic modeling and 0.620 with spline modeling. Among children with AA, temperature behaved more like a severity-related marker: maximum home temperature showed moderate apparent discrimination for complicated AA (AUC, 0.657), with no relevant gain from non-linear modeling. Bootstrap optimism correction modestly reduced the AUCs of non-linear models while preserving the qualitative pattern.

**Conclusions:**

Temperature showed outcome-dependent patterns in suspected pediatric acute appendicitis. The main finding was a non-linear association centered around the low-grade fever range for AA versus NSAP, contrasting with a more consistent association between higher temperature and complicated disease among children with confirmed AA. These findings support interpreting temperature according to the clinical outcome being evaluated, preserving it as a continuous clinical variable, and examining its functional form before reducing it to conventional fever thresholds in diagnostic scores or prediction models.

**Supplementary Information:**

The online version contains supplementary material available at 10.1007/s00383-026-06554-9.

## Introduction

Acute appendicitis (AA) has traditionally been associated with low-grade rather than high fever, particularly in uncomplicated presentations [1]. Classic scores such as the Alvarado Score and the Pediatric Appendicitis Score (PAS) were important contributions because they transformed bedside findings into structured diagnostic tools [2, 3]. However, their temperature components also illustrate a broader limitation of many clinical scores: a continuous physiological measurement is often reduced to a discrete item. In the original Alvarado score, temperature was incorporated as “slight temperature elevation,” defined as temperature ≥ 37.3 °C, whereas PAS incorporated pyrexia as a one-point component, conventionally operationalized as temperature ≥ 38.0 °C [2, 3]. These approaches made the scores practical and easy to apply, but the original derivations provide limited information on whether temperature was examined as a continuous predictor, whether alternative temperature ranges carried different diagnostic information, or whether non-linear functional forms were considered. They also predate contemporary expectations for transparent calibration assessment, internal validation, and external validation of risk models. The question is therefore not whether fever matters, but whether a conventional fever item is the best way to represent temperature in children with suspected AA.

Subsequent validation studies and systematic reviews have confirmed the clinical relevance and widespread use of these scores, but they have mainly evaluated them as constructed rather than re-examining the functional form of their individual components. A systematic review of the Alvarado score showed that its performance varied by cutoff and population, with usefulness for ruling out AA at low scores but more limited rule-in performance at higher thresholds [4]. In children, prospective validation comparing the Alvarado score and PAS supported their practical diagnostic value while again evaluating temperature as embedded within the original point-based structures [5]. Therefore, existing validation evidence supports the usefulness of appendicitis scores as pragmatic tools, but does not resolve whether temperature is optimally represented as a threshold-based item rather than as a continuous or non-linear clinical predictor. A further unresolved issue is whether maximum home temperature and ED temperature should be treated as interchangeable. Most score-based approaches use a single fever item, whereas home-reported maximum temperature and measured ED temperature may reflect different stages of the illness trajectory and may therefore carry different diagnostic or severity-related information.

## Methods

To explore whether the conventional fever item adequately captured temperature, we conducted a non-prespecified secondary exploratory analysis of the BIomarkers for the DIagnosis of Appendicitis in Pediatrics 2 (BIDIAP 2) cohort, a prospective multicenter diagnostic study of children evaluated for suspected AA across four tertiary Spanish centers between March 2024 and April 2025. The full BIDIAP 2 cohort included 717 children and classified patients as AA or non-surgical abdominal pain (NSAP) using histopathology or structured clinical follow-up as the reference standard.

Baseline sociodemographic, clinical, and laboratory variables were summarized according to appendicitis-related outcome groups. Four descriptive groups were considered: NSAP, AA, and, within AA, non-complicated AA (NCAA) and complicated AA (CAA). Continuous variables were explored visually and analytically using distributional plots, quantile-based inspection, the Shapiro–Wilk test, and Levene’s test. They were summarized as median and interquartile range. Between-group comparisons used the Wilcoxon rank-sum test, the Kruskal–Wallis test, the chi-square test, or Fisher’s exact test, as appropriate [6]. These descriptive analyses are shown in Table 1.

**Table 1:** Sociodemographic and clinical characteristics according to appendicitis-related outcome

Variable	NSAP	AA	NCAA	CAA	*P* AA vs. NSAP	*P* CAA vs. NCAA	*P* NSAP vs. NCAA vs. CAA
Age, years	10.2 [8.1–12.1]	10.3 [7.9–12.4]	10.55 [8.8–12.53]	9.5 [6.9–12.3]	0.647	**0.004**	**0.014**
Pain duration, hours	24 [12–48]	24 [12–48]	18 [9.5–30]	26.5 [20–48]	0.152	**< 0.001**	**< 0.001**
Maximum home temperature, °C	37 [36.6–38.4]	37.1 [36.7–38]	37 [36.6–37.7]	37.5 [36.85–38.5]	0.829	**< 0.001**	**0.001**
Emergency Department temperature, °C	36.5 [36–37.1]	36.8 [36.4–37.2]	36.7 [36.32–37.1]	37 [36.5–37.5]	**< 0.001**	**< 0.001**	**< 0.001**
Leukocytes, 10^9^/L	9.1 [6.93–13.2]	15.48 [12.3–18.38]	14.62 [11.56–17.29]	16.95 [13.78–20.45]	**< 0.001**	**< 0.001**	**< 0.001**
Neutrophils, 10^9^/L	5.6 [3.9–10]	12.58 [8.96–15.6]	11.55 [8.2–14.47]	14.25 [10.89–17.18]	**< 0.001**	**< 0.001**	**< 0.001**
C-reactive protein, mg/L	5 [0.6–20.4]	24.8 [8–64.68]	16.5 [5.9–38.7]	57.15 [19.1–144.15]	**< 0.001**	**< 0.001**	**< 0.001**
Sex, male/total (% male)	155/312 (49.7%)	243/404 (60.1%)	167/261 (64%)	75/142 (52.8%)	**0.005**	**0.029**	**0.002**
Peritoneal irritation, yes/total (% yes)	83/191 (43.5%)	250/306 (81.7%)	156/200 (78%)	93/105 (88.6%)	**< 0.001**	**0.023**	**< 0.001**
Vomiting, yes/total (% yes)	121/311 (38.9%)	229/401 (57.1%)	120/259 (46.3%)	109/141 (77.3%)	**< 0.001**	**< 0.001**	**< 0.001**
Hyporexia, yes/total (% yes)	148/304 (48.7%)	250/396 (63.1%)	137/256 (53.5%)	113/139 (81.3%)	**< 0.001**	**< 0.001**	**< 0.001**

AA, acute appendicitis; CAA, complicated acute appendicitis; NCAA, non-complicated acute appendicitis; NSAP, non-surgical abdominal pain; ED, Emergency Department

Continuous variables are presented as median [interquartile range], and categorical variables as n/N (%). Pairwise P-values for AA versus NSAP and CAA versus NCAA were obtained using Wilcoxon rank-sum tests for continuous variables and chi-square or Fisher’s exact tests for categorical variables, as appropriate. The global P-value for NSAP versus NCAA versus CAA was obtained using Kruskal-Wallis tests for continuous variables and chi-square or Fisher’s exact tests for categorical variables, as appropriate.

Values correspond to the complete BIDIAP 2 cohort.

Denominators may vary across variables due to missing data, including missing complication status in one patient with AA.

Percentages were calculated using the available data for each variable.

**Fig. 1:**
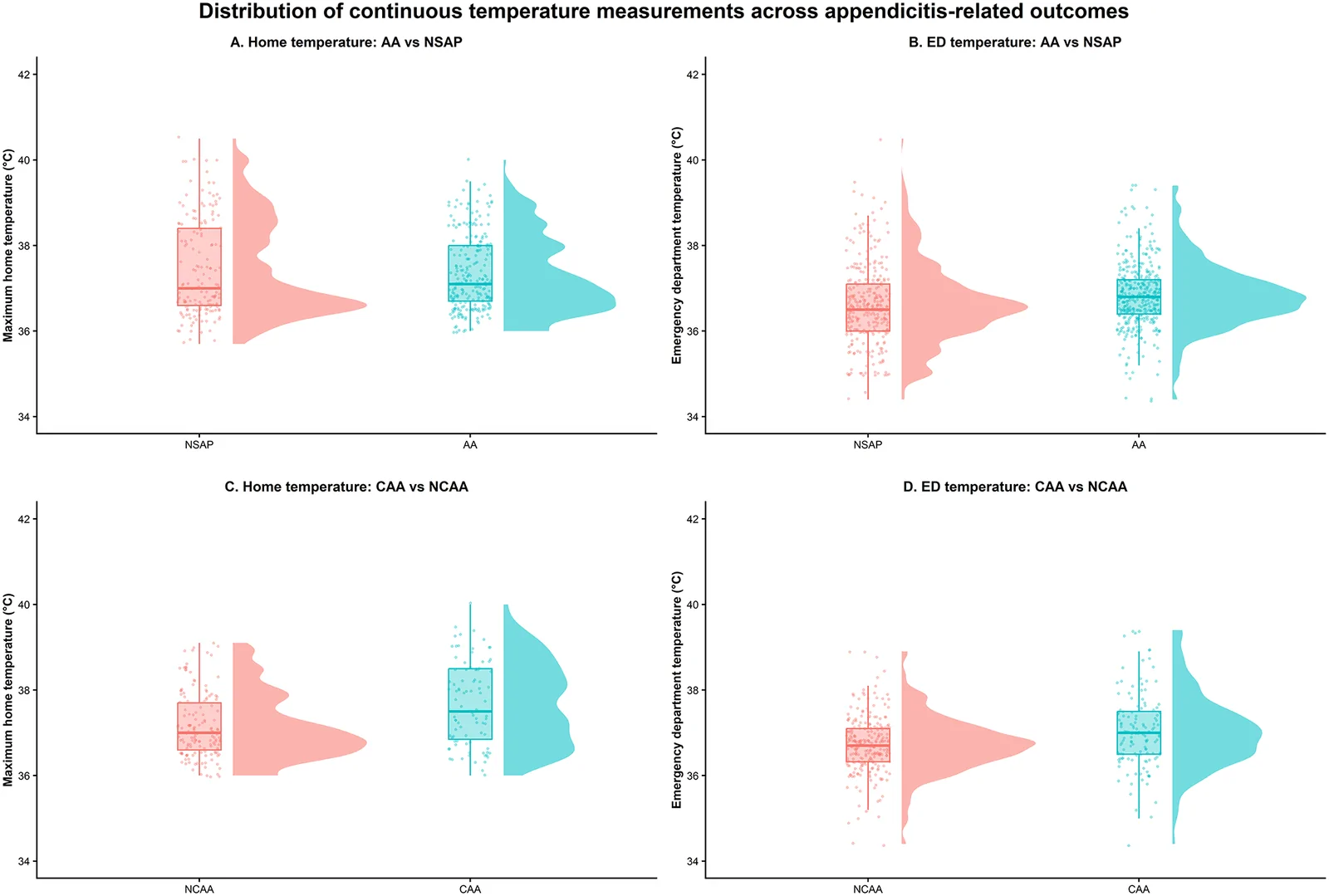
Distribution of continuous temperature measurements across appendicitis-related outcomes. Raincloud plots show maximum home temperature and Emergency Department temperature stratified by acute appendicitis (AA) versus non-surgical abdominal pain (NSAP), and by complicated acute appendicitis (CAA) versus non-complicated acute appendicitis (NCAA) among children with AA. Each plot combines individual observations, a boxplot summary, and a half-density distribution. Temperature values are expressed in degrees Celsius

Two continuous temperature measurements were evaluated: the maximum temperature reported by parents or caregivers at home before presentation to the Emergency Department (ED), and the ED temperature measured during initial hospital assessment. Both variables were analyzed as continuous quantitative measurements. Because maximum home temperature was not available for all enrolled children, analyses involving this variable were performed as complete-case exploratory analyses. To assess potential selection related to temperature availability, children with and without recorded maximum home temperature were compared across key sociodemographic, clinical, laboratory, and outcome variables; this comparison is reported in Supplementary Table 1. Their distributions were visually explored using raincloud plots [7] stratified by AA versus NSAP and, among children with AA, by CAA versus NCAA (Fig. 1).

**Fig. 2:**
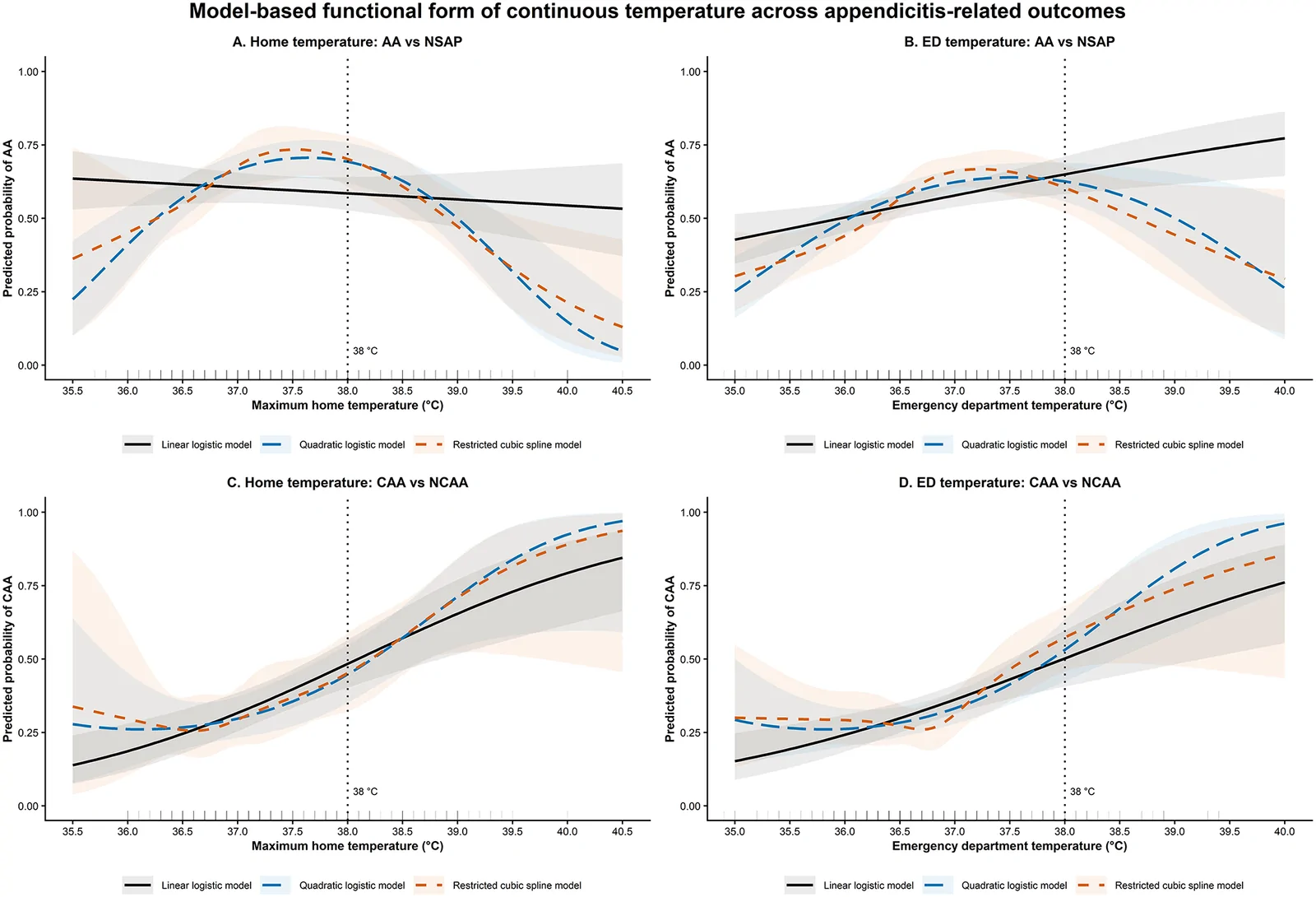
Outcome-dependent functional form of continuous temperature in suspected pediatric acute appendicitis. Predicted probabilities were estimated using single-predictor logistic regression models, with temperature entered as a linear continuous term, a quadratic continuous term, or via restricted cubic splines. Panels A and B show the associations of maximum home temperature and Emergency Department (ED) temperature, respectively, with acute appendicitis (AA) versus non-surgical abdominal pain (NSAP). In this diagnostic comparison, non-linear models suggested an inverted U-shaped relationship, with the highest estimated probability of AA occurring in the low-grade fever range rather than above the conventional 38 °C fever threshold. Panels C and D show the corresponding associations with complicated acute appendicitis (CAA) versus non-complicated acute appendicitis (NCAA) among children with AA. In this severity-related comparison, temperature behaved more like a marker of disease severity, with higher values associated with greater predicted probability of CAA. The dotted vertical line indicates 38 °C. Curves and AUCs should be interpreted as exploratory apparent in-sample estimates, not as validated prediction models.

**Fig. 3:**
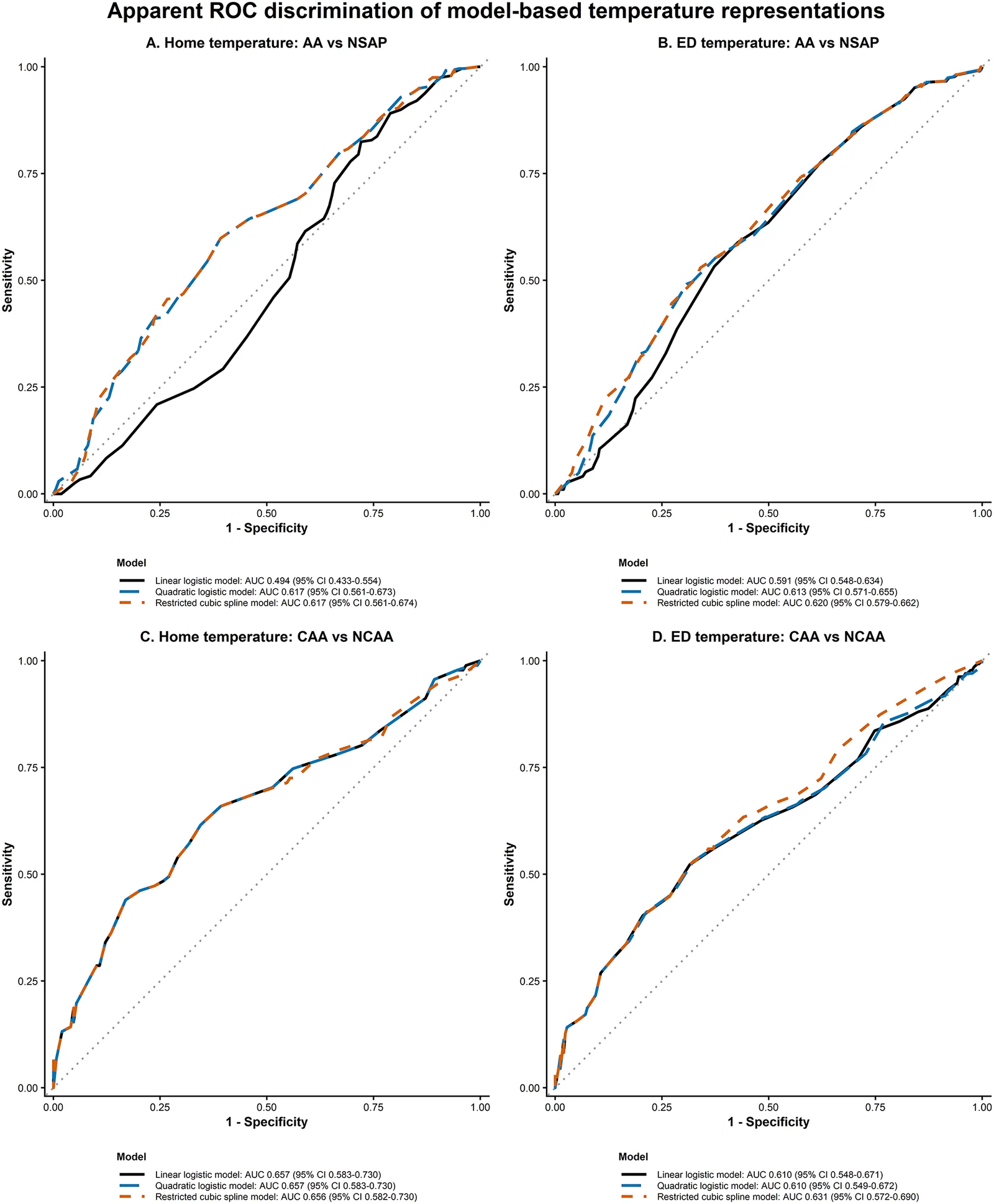
Apparent ROC discrimination of logistic temperature model specifications. Receiver operating characteristic curves were constructed from predicted probabilities generated by single-predictor logistic models in which temperature was represented as a linear continuous term, a quadratic term, or with restricted cubic splines. Panels A and B show apparent discrimination for AA versus NSAP using maximum home temperature and Emergency Department temperature, respectively. Panels C and D show the corresponding analyses for CAA versus NCAA among children with AA. Because each curve was derived from a model fitted and evaluated in the same dataset, all AUCs should be interpreted as apparent in-sample estimates

We fitted single-predictor logistic regression models using temperature as a linear continuous term, a quadratic continuous term, and restricted cubic splines (RCS) with five knots placed at the 5th, 27.5th, 50th, 72.5th, and 95th sample percentiles [8]. In these models, “linear” refers to the functional form of temperature on the logit scale, not to ordinary least-squares linear regression. Model-derived predicted probabilities were plotted to visualize the functional form of the temperature-outcome association (Fig. 2). Receiver operating characteristic (ROC) curves were constructed directly from the predicted probabilities generated by the logistic regression models, and apparent AUCs with 95% confidence intervals (CIs) were estimated (Fig. 3). Likelihood-ratio tests compared the linear model with the quadratic and RCS specifications. Because all models were fitted and evaluated on the same data, we additionally performed a bootstrap optimism correction with 500 resamples. Apparent calibration intercept, calibration slope, and Brier score were calculated [9] only as descriptive in-sample performance indices. Because the same data were used for model fitting and assessment, calibration intercepts and slopes were not interpreted as evidence of model validity.

Because the main objective was to examine the functional form of temperature rather than to develop a multivariable diagnostic model, the primary analyses used single-predictor models. As exploratory adjusted sensitivity analyses, we additionally refitted the temperature models after adjustment for age and pain duration. A second extended sensitivity analysis additionally adjusted for C-reactive protein, modeled as log(1 + CRP), as a representative inflammatory marker. The adjusted sensitivity analyses are reported in Supplementary Table 3.

Additional exploratory analyses were performed among children presenting within 24 h of pain onset and in a direct comparison of NSAP versus NCAA. These exploratory analyses were performed for two reasons. First, the subgroup of children presenting within 24 h of pain onset was examined because body temperature is a time-dependent clinical variable, and the relationship between fever and appendicitis may differ in early presentations before the inflammatory response has fully evolved. Second, a direct comparison of NSAP versus NCAA was used to assess whether the temperature signal was present specifically in NCAA, rather than being mainly driven by complicated appendicitis, in which systemic inflammation and fever are expected to be more pronounced.

Literature-based temperature cutoffs were also evaluated by applying the Alvarado-associated 37.3 °C threshold and the PAS/conventional 38.0 °C threshold directly to the observed temperature values. For these threshold analyses, sensitivity, specificity, positive predictive value, negative predictive value, positive and negative likelihood ratios, Youden index, and accuracy were calculated and reported in Supplementary Table 2. All analyses were performed using R version 4.6.0, and two-sided p-values < 0.05 were considered statistically significant.

## Results

Baseline sociodemographic and clinical characteristics are summarized in Table 1. Age was similar between AA and NSAP but differed between CAA and NCAA. Median pain duration was longer in CAA than in NCAA. Male sex, peritoneal irritation, ED temperature, leukocytes, neutrophils, C-reactive protein, vomiting, and hyporexia differed significantly between AA and NSAP, as well as across the three mutually exclusive groups: NSAP, NCAA, and CAA. Maximum home temperature did not differ meaningfully between AA and NSAP on crude group summaries, but was higher in CAA than in NCAA. Children with recorded maximum home temperature were not fully exchangeable with those without this measurement. Compared with children without recorded home temperature, they were younger, had longer pain duration, higher ED temperature, higher leukocyte and neutrophil counts, and higher C-reactive protein levels. They also more frequently presented with vomiting and hyporexia, and a higher proportion were ultimately diagnosed with AA. No statistically significant differences were observed for sex, peritoneal irritation, or for the proportion of CAA among children with AA. These findings, shown in Supplementary Table 1, indicate that maximum home temperature availability was clinically patterned rather than completely random. Therefore, analyses involving maximum home temperature should be interpreted as complete-case exploratory findings potentially affected by selection bias. Raincloud plots showed substantial overlap between AA and NSAP for both temperature measurements. For maximum home temperature, NSAP also showed high-temperature values, including density in the 39–40 °C range, while AA did not display a uniformly more extended febrile tail. ED temperature showed even greater overlap, with only a modest upward shift in AA. By contrast, among patients with AA, CAA was more clearly shifted toward higher temperatures than NCAA, especially for maximum home temperature; however, overlap remained considerable, particularly for ED temperature (Fig. 1).

In the AA-versus-NSAP analysis, maximum home temperature data were available for 400 children, including 239 with AA and 161 with NSAP. A logistic model with temperature entered as a linear continuous term showed no apparent discrimination (AUC, 0.494; 95% CI, 0.433–0.554). In contrast, non-linear specifications yielded higher apparent AUCs for both the quadratic model (AUC, 0.617; 95% CI, 0.561–0.673) and the RCS model (AUC, 0.617; 95% CI, 0.561–0.674). The quadratic curve for maximum home temperature reached its maximum within the low-grade fever range, around 37.6 °C in the apparent model, and likelihood-ratio testing supported departure from linearity for both the quadratic and RCS specifications (both *p* < 0.001). ED temperature showed a similar but weaker pattern in 698 children, including 389 with AA and 309 with NSAP, with the quadratic curve peaking at approximately 37.5 °C and AUC increasing from 0.591 (95% CI, 0.548–0.634) in the logistic model with a linear temperature term to 0.613 (95% CI, 0.571–0.655) with quadratic modeling and 0.620 (95% CI, 0.579–0.662) with RCS. The departure from linearity was again supported for both non-linear ED temperature specifications (*p* < 0.001 for both). These model-based functional forms are shown in Fig. 2, and their corresponding apparent ROC curves are shown in Fig. 3.

In the CAA-versus-NCAA analysis, maximum home temperature was available in 239 children with AA, including 91 with CAA and 148 with NCAA. In contrast to the AA-versus-NSAP comparison, temperature behaved more like a severity-related marker. Maximum home temperature showed moderate apparent discrimination in the linear model (AUC, 0.657; 95% CI, 0.583–0.730), with no relevant gain after quadratic modeling (AUC, 0.657; 95% CI, 0.583–0.730) or RCS (AUC, 0.656; 95% CI, 0.582–0.730). Likelihood-ratio tests did not support a departure from linearity for maximum home temperature in this comparison (linear versus quadratic: *p* = 0.214; linear versus RCS: *p* = 0.668). ED temperature showed weaker discrimination for CAA in the linear model (AUC, 0.610; 95% CI, 0.548–0.671), with no gain after quadratic modeling (AUC, 0.610; 95% CI, 0.549–0.672) and only a small apparent increase using RCS (AUC, 0.631; 95% CI, 0.572–0.690). For ED temperature, the quadratic model showed statistical evidence of departure from linearity (*p* = 0.028), whereas the RCS comparison did not (*p* = 0.097). The functional-form plots illustrate this contrast: temperature showed a non-monotonic low-grade fever pattern for AA versus NSAP, whereas for CAA versus NCAA it behaved more consistently as a severity-related marker (Fig. 2). ROC analyses supported the same interpretation, with greater apparent benefit from non-linear modeling in the AA-versus-NSAP comparison than in the CAA-versus-NCAA comparison (Fig. 3).

Bootstrap optimism correction modestly reduced the AUCs of the non-linear models while preserving the qualitative pattern between AA and NSAP, particularly for maximum home temperature. For maximum home temperature in the AA-versus-NSAP comparison, the optimism-corrected AUC was 0.610 for the quadratic model and 0.602 for the RCS model, compared with 0.477 for the linear model. For ED temperature in the same comparison, the corresponding optimism-corrected AUCs were 0.609 for the quadratic model, 0.613 for the RCS model, and 0.591 for the linear model. Apparent calibration intercepts and slopes were 0 and 1, respectively, as expected for models assessed in the same data used for fitting; these calibration metrics were therefore interpreted descriptively rather than as evidence of external model validity.

Exploratory adjusted sensitivity analyses are reported in Supplementary Table 3. After adjustment for age and pain duration, the non-linear temperature pattern in the AA-versus-NSAP comparison was preserved: for maximum home temperature, the AUC increased from 0.555 in the linear adjusted model to 0.636 in both the quadratic and RCS adjusted models; for ED temperature, the AUC increased from 0.601 to 0.626 and 0.628, respectively. In the CAA-versus-NCAA comparison, adjusted AUCs were higher overall, consistent with the contribution of age and pain duration to severity discrimination, but the incremental difference between linear and non-linear temperature specifications was smaller. In the extended models additionally adjusted for log(1 + C-reactive protein), overall AUCs increased further, but non-linear temperature terms added only limited additional apparent discrimination, particularly for CAA versus NCAA. These models should be interpreted as sensitivity analyses rather than as multivariable diagnostic prediction models, because their purpose was to evaluate attenuation or preservation of the temperature pattern rather than to estimate the independent clinical utility of temperature.

Exploratory sensitivity analyses supported the same interpretation. Among children presenting within 24 h of pain onset, non-linear modeling of maximum home temperature again yielded higher optimism-corrected AUCs than the linear specification for AA versus NSAP, with corrected AUCs of 0.653 for the quadratic model and 0.639 for the restricted cubic spline model, compared with 0.532 for the linear model. Similarly, when NSAP was compared directly with NCAA, the low-grade fever pattern was preserved: for maximum home temperature, the optimism-corrected AUCs were 0.638 for the quadratic model and 0.624 for the restricted cubic spline model, compared with 0.538 for the linear model. A complete-case sensitivity analysis restricted to children with both home and ED temperature available produced almost identical findings for maximum home temperature, suggesting that the main maximum-home-temperature pattern was not materially altered when analyses were restricted to children with both measurements available.

In supplementary threshold analyses, the Alvarado-associated 37.3 °C cutoff and the PAS/conventional 38.0 °C cutoff were applied directly to the observed temperature values [2, 3]. These analyses showed poor diagnostic performance for distinguishing AA from NSAP, with likelihood ratios close to 1 across both home and ED temperature thresholds. In contrast, the 38.0 °C threshold behaved more like a specific but insensitive marker of CAA, particularly for ED temperature, where the positive likelihood ratio (LR+) exceeded 5 despite very low sensitivity. Full threshold-based diagnostic performance metrics, including sensitivity, specificity, predictive values, likelihood ratios, Youden index, and accuracy, are reported in Supplementary Table 2.

## Discussion

This exploratory secondary analysis evaluated whether two routinely available continuous temperature measurements—maximum home temperature before ED presentation and temperature measured at ED admission—carry clinically relevant information in children with suspected AA that may be obscured when temperature is reduced to conventional binary fever thresholds. Rather than deriving a new score or proposing a new cutoff, this analysis examined the functional form of the temperature–outcome association across two clinically distinct questions: diagnostic discrimination between AA and NSAP, and severity stratification between CAA and NCAA. This distinction is central to the clinical interpretation of the findings, because the same temperature value may not carry the same meaning when the clinician is deciding whether appendicitis is present as when the clinician is assessing disease severity among children with confirmed AA. In doing so, it provides a clinical and methodological illustration of why continuous variables should be explored on their original scale before being dichotomized into score components, in line with contemporary recommendations for handling continuous predictors [8–11].

Although the analysis used flexible modeling approaches, the clinical question was intentionally practical: whether a routinely available bedside variable is adequately represented by a binary fever threshold. The quadratic and spline models were used to examine the functional form of the temperature–outcome association, not to develop a complex computational tool for immediate clinical deployment. The findings, therefore, have direct relevance for clinicians involved in the assessment of suspected pediatric acute appendicitis, because they suggest that maximum home temperature and ED temperature may carry different information and should not automatically be treated as interchangeable binary fever items in future appendicitis scores or model updates.

The main finding was that temperature carried different information in two distinct clinical questions. When discriminating AA from NSAP, maximum home temperature behaved as a non-linear diagnostic marker with modest apparent discrimination, whereas among children with confirmed AA, higher temperatures more consistently marked complicated disease. In the AA-versus-NSAP comparison, maximum home temperature was poorly represented by a simple monotonic gradient: the linear model showed no apparent discrimination, whereas quadratic and RCS models suggested that the most informative range lay within the low-grade fever range. This pattern is clinically plausible because AA in children has traditionally been associated with low-grade rather than high fever, especially in uncomplicated or earlier presentations [1]. Conversely, in the CAA-versus-NCAA comparison, temperature behaved more like a severity-related marker, with higher values associated with complicated disease. Therefore, the same physiological variable should not be assigned a single invariant meaning across all appendicitis-related decisions. A temperature value that provides limited diagnostic discrimination between AA and NSAP may still contribute to severity stratification among children with confirmed AA.

An additional interpretive consideration is that the composition of the NSAP comparator may partly shape the observed functional form. NSAP is a heterogeneous category that may include both predominantly afebrile and febrile non-appendicitis conditions. Therefore, the descending limb of the inverted U-shaped association at higher temperatures could partly reflect a greater relative representation of febrile non-appendicitis diagnoses in that range, rather than an intrinsic non-monotonic relationship between temperature and AA. Under this interpretation, the low-grade fever peak would represent the temperature range in which AA was comparatively more frequent than both afebrile and highly febrile mimics within this particular cohort. The precise shape of the association may consequently depend on the diagnostic composition of the comparator population and may not transport unchanged to other clinical settings. Future studies could stratify non-appendicitis presentations according to their underlying diagnoses or febrile phenotype to determine whether the observed pattern reflects a reproducible temperature–appendicitis association or is partly driven by the distribution of competing diagnoses.

The supplementary threshold analyses reinforce this distinction. Conventional temperature cutoffs remain useful clinical benchmarks, but they should not be interpreted as definitive representations of the temperature–risk relationship. The Alvarado-associated 37.3 °C cutoff and the PAS/conventional 38.0 °C cutoff were straightforward to evaluate on the raw temperature scale [2, 3], yet both performed poorly for distinguishing AA from NSAP, with likelihood ratios close to 1. In contrast, the 38.0 °C cutoff behaved more like a specific but insensitive marker of CAA, particularly for ED temperature. This pattern supports the central methodological point of the study: a rigid cutoff is poorly suited to representing a continuous variable when the underlying association is non-linear or non-monotonic. In the AA-versus-NSAP comparison, risk appeared to peak within an intermediate low-grade fever range rather than above a single conventional fever threshold, meaning that simple dichotomization may obscure rather than clarify the clinically relevant signal.

These findings should not be interpreted as the derivation of a new diagnostic score, as a recommendation to modify existing fever cutoffs, or as evidence that a specific temperature range should replace existing score components. Although non-linear modeling improved discrimination compared with the linear specification, overall performance remained modest and does not support the use of temperature as an independent diagnostic predictor. Rather, the findings show that a familiar bedside variable may contain outcome-dependent information that is lost when it is forced into a binary item. Classic AA scores such as the Alvarado score and the Pediatric Appendicitis Score remain important pragmatic tools [2, 3], and subsequent validation studies have supported their clinical usefulness [4, 5]. However, these scores were developed before current expectations regarding functional-form assessment, calibration, internal validation, and external validation of prediction models. The present findings, therefore, do not challenge the usefulness of these scores as clinical tools but highlight that their individual continuous components may deserve re-examination using contemporary modeling approaches.

One clinically relevant interpretation is that maximum home temperature and ED temperature may capture different stages of the same illness trajectory. In routine pediatric assessment, parents often measure temperature repeatedly as symptoms evolve before hospital presentation, whereas ED temperature is a single time-point measurement obtained after variable intervals of pain, possible antipyretic administration, transport, waiting time, and initial clinical care. Therefore, a normal or only slightly elevated ED temperature may coexist with a preceding febrile pattern at home. In the present study, this distinction was clinically meaningful because maximum home temperature and ED temperature showed different degrees of overlap and different apparent functional forms across the evaluated outcomes. The practical implication is that temperature history should be recorded with greater granularity, including the maximum value reported at home, timing in relation to symptom onset, and antipyretic exposure when available, rather than being reduced to a single binary fever item. This is especially relevant for future appendicitis scores or model updates, where home-reported maximum temperature and ED temperature should be considered as related but non-interchangeable variables. At the bedside, these findings support a more nuanced interpretation of temperature as part of the overall clinical picture, particularly when distinguishing diagnostic assessment of AA from severity assessment among children with confirmed AA.

This study has several limitations. First, the analysis was exploratory and not prespecified in the original BIDIAP 2 protocol; therefore, all findings should be interpreted as hypothesis-generating. Second, the interpretation of home-temperature analyses is limited by the incomplete and clinically patterned availability of maximum home temperature. Children with recorded maximum home temperature differed systematically from those without this measurement, including differences in pain duration, ED temperature, inflammatory markers, vomiting, hyporexia, and final AA diagnosis. This pattern, detailed in Supplementary Table 1, suggests that missingness was unlikely to be completely at random and that complete-case analyses may have introduced selection bias. Therefore, the home-temperature findings should be viewed as clinically informative but exploratory and require confirmation in studies with prospective, systematic recording of home temperature, timing of measurement, and antipyretic exposure. Third, the primary models were intentionally restricted to single temperature predictors to examine functional form and should not be interpreted as estimating an independent diagnostic effect of temperature. Although exploratory sensitivity analyses adjusted for age, pain duration, and C-reactive protein were performed, the study was not designed to develop a full multivariable diagnostic model. Other clinically relevant variables, including leukocyte count, neutrophil count, imaging findings, and prior antipyretic administration, may alter the apparent contribution of temperature. Inflammatory markers may also partly reflect the same underlying inflammatory process captured by fever, making the distinction between confounding, mediation, and incremental prediction clinically complex. Therefore, the present findings should be interpreted as a functional-form analysis of temperature rather than as evidence that temperature independently predicts appendicitis or complicated disease after accounting for the full clinical and laboratory profile. Fourth, no external validation was performed. Although bootstrap optimism correction was used to quantify internal optimism, it does not replace validation in an independent cohort. The reported AUCs, calibration metrics, and functional-form patterns should therefore be interpreted as internal exploratory findings requiring confirmation before being used for clinical prediction or score updating.

Nevertheless, these limitations are balanced by several strengths. This study was based on a prospective multicenter cohort and evaluated two clinically distinct temperature measurements: maximum temperature recorded at home and temperature measured in the Emergency Department. The analysis also preserved the distinction between diagnostic discrimination for AA versus NSAP and severity stratification for CAA versus NCAA, avoiding the common error of treating these as interchangeable clinical questions. Methodologically, the study employed a robust, layered analytical strategy that combined distributional visualization, functional-form assessment, ROC analysis, likelihood-ratio testing, bootstrap optimism correction, threshold-based diagnostic metrics, and sensitivity analyses. A particularly relevant strength is its exploratory use of non-linear modeling to examine temperature as a continuous variable, rather than assuming linearity or imposing conventional fever cutoffs. This is important because, in pediatric appendicitis, temperature is commonly operationalized as a categorical score component, whereas its continuous functional form and the distinction between maximum home temperature and ED temperature have received limited attention. This approach allowed the study to identify clinically plausible outcome-dependent patterns that would have been obscured by conventional dichotomization.

Future AA scores or model updates should explicitly evaluate whether low-grade fever carries risk-informative value as part of the continuous temperature function, rather than assuming that only temperatures above a conventional fever threshold contribute diagnostic information. Such an approach would require formal model development, calibration assessment, optimism correction, and external validation before being translated into any point-based score or clinical decision rule. This distinction is aligned with contemporary modeling recommendations, which favor preserving continuous predictors, examining functional form, and avoiding unnecessary categorization when developing or evaluating clinical prediction models [10, 11].

## Supplementary Information

Below is the link to the electronic supplementary material.

Supplementary Material 1

## Data Availability

The data used to carry out this study are available upon reasonable request from the corresponding author.
