# Supplementary material for: Temperature patterns in suspected pediatric acute appendicitis
Source: Pediatr Surg Int. 2026 Aug 26;42(1):365. doi: 10.1007/s00383-026-06554-9 (PMC13518472; doi:10.1007/s00383-026-06554-9)
Supplement: Supplementary file 1 [file 383_2026_6554_MOESM1_ESM.docx]

**Supplementary Table 1. Comparison of children with and without available maximum home temperature.**

| **Variable** | **Home temperature available (n=400)** | **Home temperature unavailable (n=317)** | **P value** |
| --- | --- | --- | --- |
| Age, years | 10 [7.5-12.1] | 10.8 [8.85-12.5] | **0.001** |
| Pain duration, hours | 24 [12-48] | 20 [12-36] | **<0.001** |
| Emergency Department temperature, °C | 36.8 [36.5-37.4] | 36.4 [36-36.8] | **<0.001** |
| Leukocytes, 10^9^/L | 13.7 [9.3-17.7] | 12.04 [8.3-16.2] | **0.004** |
| Neutrophils, 10^9^/L | 10.75 [5.98-14.9] | 8.85 [4.94-13.15] | **0.001** |
| C-reactive protein, mg/L | 24.8 [6.4-71.8] | 6 [1.3-22] | **<0.001** |
| Sex, male/total (% male) | 234/400 (58.5%) | 164/316 (51.9%) | 0.078 |
| Peritoneal irritation, yes/total (% yes) | 181/274 (66.1%) | 152/223 (68.2%) | 0.620 |
| Vomiting, yes/total (% yes) | 213/399 (53.4%) | 137/313 (43.8%) | **0.011** |
| Hyporexia, yes/total (% yes) | 243/393 (61.8%) | 155/307 (50.5%) | **0.003** |
| Acute appendicitis diagnosis, yes/total (% yes) | 239/400 (59.8%) | 165/317 (52.1%) | **0.039** |
| Complicated acute appendicitis among children with acute appendicitis, yes/total (% yes) | 91/239 (38.1%) | 51/164 (31.1%) | 0.150 |

*Continuous variables are summarized as median [interquartile range]. Categorical variables are summarized as n/N (%).*

Children with and without recorded maximum home temperature were compared across key sociodemographic, clinical, laboratory, and outcome variables to assess potential selection related to temperature availability.

**Supplementary Table 2. Diagnostic performance associated with literature-based temperature cutoffs.**

| **Comparison** | **Temperature variable** | **Cutoff source** | **Cutoff, °C** | **N** | **Events** | **Non-events** | **TP** | **FP** | **TN** | **FN** | **Sensitivity** | **Specificity** | **PPV** | **NPV** | **LR+** | **LR-** | **Youden** | **Accuracy** |
| --- | --- | --- | --- | --- | --- | --- | --- | --- | --- | --- | --- | --- | --- | --- | --- | --- | --- | --- |
| AA vs NSAP | Maximum home temperature | Alvarado threshold | ≥37.3 °C | 400 | 239 | 161 | 107 | 70 | 91 | 132 | 0.448 | 0.565 | 0.605 | 0.408 | 1.03 | 0.977 | 0.013 | 0.495 |
| AA vs NSAP | Maximum home temperature | PAS/conventional fever threshold | ≥38.0 °C | 400 | 239 | 161 | 65 | 55 | 106 | 174 | 0.272 | 0.658 | 0.542 | 0.379 | 0.796 | 1.106 | -0.07 | 0.428 |
| AA vs NSAP | Emergency Department temperature | Alvarado threshold | ≥37.3 °C | 698 | 389 | 309 | 87 | 58 | 251 | 302 | 0.224 | 0.812 | 0.6 | 0.454 | 1.192 | 0.956 | 0.036 | 0.484 |
| AA vs NSAP | Emergency Department temperature | PAS/conventional fever threshold | ≥38.0 °C | 698 | 389 | 309 | 26 | 28 | 281 | 363 | 0.067 | 0.909 | 0.481 | 0.436 | 0.738 | 1.026 | -0.024 | 0.44 |
| CAA vs NCAA | Maximum home temperature | Alvarado threshold | ≥37.3 °C | 239 | 91 | 148 | 56 | 51 | 97 | 35 | 0.615 | 0.655 | 0.523 | 0.735 | 1.786 | 0.587 | 0.271 | 0.64 |
| CAA vs NCAA | Maximum home temperature | PAS/conventional fever threshold | ≥38.0 °C | 239 | 91 | 148 | 40 | 25 | 123 | 51 | 0.44 | 0.831 | 0.615 | 0.707 | 2.602 | 0.674 | 0.271 | 0.682 |
| CAA vs NCAA | Emergency Department temperature | Alvarado threshold | ≥37.3 °C | 388 | 134 | 254 | 45 | 42 | 212 | 89 | 0.336 | 0.835 | 0.517 | 0.704 | 2.031 | 0.796 | 0.17 | 0.662 |
| CAA vs NCAA | Emergency Department temperature | PAS/conventional fever threshold | ≥38.0 °C | 388 | 134 | 254 | 19 | 7 | 247 | 115 | 0.142 | 0.972 | 0.731 | 0.682 | 5.145 | 0.883 | 0.114 | 0.686 |

*AA, acute appendicitis; CAA, complicated acute appendicitis; LR-, negative likelihood ratio; LR+, positive likelihood ratio; NCAA, non-complicated acute appendicitis; NPV, negative predictive value; NSAP, non-surgical abdominal pain; PAS, Pediatric Appendicitis Score; PPV, positive predictive value; TP, true positives; FP, false positives; TN, true negatives; FN, false negatives*

Diagnostic performance associated with literature-based temperature cutoffs from the Alvarado Score and Pediatric Appendicitis Score across the evaluated clinical scenarios. The Alvarado-associated 37.3 °C threshold and the PAS/conventional 38.0 °C threshold were applied directly to observed temperature values. Diagnostic performance was summarized using sensitivity, specificity, positive and negative predictive values, positive and negative likelihood ratios, Youden index, and accuracy.

**Supplementary Table 3. Exploratory adjusted sensitivity analyses**

| **Comparison** | **Temperature variable** | **Adjustment** | **N** | **Events** | **Non-events** | **Model** | **AUC** | **95% CI** |
| --- | --- | --- | --- | --- | --- | --- | --- | --- |
| AA vs NSAP | Maximum home temperature | Adjusted for age and pain duration | 395 | 236 | 159 | Linear adjusted logistic model | 0.555 | 0.497-0.614 |
| AA vs NSAP | Maximum home temperature | Adjusted for age and pain duration | 395 | 236 | 159 | Quadratic adjusted logistic model | 0.636 | 0.581-0.692 |
| AA vs NSAP | Maximum home temperature | Adjusted for age and pain duration | 395 | 236 | 159 | Restricted cubic spline adjusted logistic model | 0.636 | 0.581-0.692 |
| AA vs NSAP | Emergency Department temperature | Adjusted for age and pain duration | 682 | 383 | 299 | Linear adjusted logistic model | 0.601 | 0.557-0.644 |
| AA vs NSAP | Emergency Department temperature | Adjusted for age and pain duration | 682 | 383 | 299 | Quadratic adjusted logistic model | 0.626 | 0.583-0.668 |
| AA vs NSAP | Emergency Department temperature | Adjusted for age and pain duration | 682 | 383 | 299 | Restricted cubic spline adjusted logistic model | 0.628 | 0.586-0.670 |
| CAA vs NCAA | Maximum home temperature | Adjusted for age and pain duration | 236 | 89 | 147 | Linear adjusted logistic model | 0.751 | 0.687-0.815 |
| CAA vs NCAA | Maximum home temperature | Adjusted for age and pain duration | 236 | 89 | 147 | Quadratic adjusted logistic model | 0.768 | 0.708-0.829 |
| CAA vs NCAA | Maximum home temperature | Adjusted for age and pain duration | 236 | 89 | 147 | Restricted cubic spline adjusted logistic model | 0.765 | 0.704-0.826 |
| CAA vs NCAA | Emergency Department temperature | Adjusted for age and pain duration | 382 | 131 | 251 | Linear adjusted logistic model | 0.705 | 0.648-0.762 |
| CAA vs NCAA | Emergency Department temperature | Adjusted for age and pain duration | 382 | 131 | 251 | Quadratic adjusted logistic model | 0.713 | 0.657-0.769 |
| CAA vs NCAA | Emergency Department temperature | Adjusted for age and pain duration | 382 | 131 | 251 | Restricted cubic spline adjusted logistic model | 0.718 | 0.664-0.772 |
| AA vs NSAP | Maximum home temperature | Adjusted for age, pain duration, and log(1 + CRP) | 392 | 233 | 159 | Linear adjusted logistic model | 0.746 | 0.695-0.797 |
| AA vs NSAP | Maximum home temperature | Adjusted for age, pain duration, and log(1 + CRP) | 392 | 233 | 159 | Quadratic adjusted logistic model | 0.772 | 0.724-0.820 |
| AA vs NSAP | Maximum home temperature | Adjusted for age, pain duration, and log(1 + CRP) | 392 | 233 | 159 | Restricted cubic spline adjusted logistic model | 0.771 | 0.723-0.819 |
| AA vs NSAP | Emergency Department temperature | Adjusted for age, pain duration, and log(1 + CRP) | 676 | 378 | 298 | Linear adjusted logistic model | 0.756 | 0.719-0.794 |
| AA vs NSAP | Emergency Department temperature | Adjusted for age, pain duration, and log(1 + CRP) | 676 | 378 | 298 | Quadratic adjusted logistic model | 0.77 | 0.733-0.806 |
| AA vs NSAP | Emergency Department temperature | Adjusted for age, pain duration, and log(1 + CRP) | 676 | 378 | 298 | Restricted cubic spline adjusted logistic model | 0.776 | 0.740-0.812 |
| CAA vs NCAA | Maximum home temperature | Adjusted for age, pain duration, and log(1 + CRP) | 233 | 89 | 144 | Linear adjusted logistic model | 0.799 | 0.739-0.858 |
| CAA vs NCAA | Maximum home temperature | Adjusted for age, pain duration, and log(1 + CRP) | 233 | 89 | 144 | Quadratic adjusted logistic model | 0.797 | 0.738-0.856 |
| CAA vs NCAA | Maximum home temperature | Adjusted for age, pain duration, and log(1 + CRP) | 233 | 89 | 144 | Restricted cubic spline adjusted logistic model | 0.797 | 0.738-0.857 |
| CAA vs NCAA | Emergency Department temperature | Adjusted for age, pain duration, and log(1 + CRP) | 377 | 129 | 248 | Linear adjusted logistic model | 0.75 | 0.696-0.804 |
| CAA vs NCAA | Emergency Department temperature | Adjusted for age, pain duration, and log(1 + CRP) | 377 | 129 | 248 | Quadratic adjusted logistic model | 0.753 | 0.700-0.807 |
| CAA vs NCAA | Emergency Department temperature | Adjusted for age, pain duration, and log(1 + CRP) | 377 | 129 | 248 | Restricted cubic spline adjusted logistic model | 0.754 | 0.702-0.807 |

*CRP was modeled as log(1 + CRP). AUC, area under the receiver operating characteristic curve; CI, confidence interval; CRP, C-reactive protein; AA, acute appendicitis; CAA, complicated acute appendicitis; NCAA, non-complicated acute appendicitis; NSAP, non-surgical abdominal pain.*

Exploratory adjusted sensitivity analyses of apparent discrimination in temperature functional-form models.
